# Race and BMI modify associations of calcium and vitamin D intake with prostate cancer

**DOI:** 10.1186/s12885-017-3060-8

**Published:** 2017-01-19

**Authors:** Ken Batai, Adam B. Murphy, Maria Ruden, Jennifer Newsome, Ebony Shah, Michael A. Dixon, Elizabeth T. Jacobs, Courtney M. P. Hollowell, Chiledum Ahaghotu, Rick A. Kittles

**Affiliations:** 1 Division of Urology, Department of Surgery, The University of Arizona College of Medicine, University of Arizona Cancer Center, 1515 N. Campbell Ave, P.O. Box 245024, Tucson, AZ 85724 USA; 2 Department of Urology, Feinberg School of Medicine, Northwestern University, 303 E. Chicago Ave, Chicago, IL 60611 USA; 3 Department of Medicine, University of Illinois at Chicago, 840 South Wood Street, Suite 1020 N (MC 787), Chicago, IL 60612 USA; 4 Center for Clinical and Translational Science, University of Illinois at Chicago, 914 S Wood Street (MC 595), Chicago, IL 60612 USA; 5 Division of Epidemiology and Biostatistics, Mel and Enid Zuckerman College of Public Health, University of Arizona, 1295 N. Martin Ave, PO Box 245210, Tucson, AZ 85724 USA; 6 Division of Urology, Cook County Health and Hospitals System, 1900 W. Polk Ave., Suite 465, Chicago, IL 60612 USA; 7 Carney Hospital-Steward Health System, 2100 Dorchester Avenue, Dorchester, MA 02124 USA

**Keywords:** African Americans, Calcium intake, Vitamin D intake, Prostate cancer

## Abstract

**Background:**

African Americans have disproportionately higher burden of prostate cancer compared to European Americans. However, the cause of prostate cancer disparities is still unclear. Several roles have been proposed for calcium and vitamin D in prostate cancer pathogenesis and progression, but epidemiologic studies have been conducted mainly in European descent populations. Here we investigated the association of calcium and vitamin D intake with prostate cancer in multiethnic samples.

**Methods:**

A total of 1,657 prostate cancer patients who underwent screening and healthy controls (888 African Americans, 620 European Americans, 111 Hispanic Americans, and 38 others) from Chicago, IL and Washington, D.C. were included in this study. Calcium and vitamin D intake were evaluated using food frequency questionnaire. We performed unconditional logistic regression analyses adjusting for relevant variables.

**Results:**

In the pooled data set, high calcium intake was significantly associated with higher odds for aggressive prostate cancer (OR_Quartile 1 vs. Quartile 4_ = 1.98, 95% C.I.: 1.01–3.91), while high vitamin D intake was associated with lower odds of aggressive prostate cancer (OR_Quartile 1 vs. Quartile 4_ = 0.38, 95% C.I.: 0.18–0.79). In African Americans, the association between high calcium intake and aggressive prostate cancer was statistically significant (OR_Quartile 1 vs. Quartile 4_ = 4.28, 95% C.I.: 1.70–10.80). We also observed a strong inverse association between total vitamin D intake and prostate cancer in African Americans (OR_Quartile 1 vs. Quartile 4_ = 0.06, 95% C.I.: 0.02–0.54). In European Americas, we did not observe any significant associations between either calcium or vitamin D intake and prostate cancer. In analyses stratifying participants based on Body Mass Index (BMI), we observed a strong positive association between calcium and aggressive prostate cancer and a strong inverse association between vitamin D intake and aggressive prostate cancer among men with low BMI (<27.8 kg/m^2^), but not among men with high BMI (≥27.8 kg/m^2^). Interactions of race and BMI with vitamin D intake were significant (*P*_Interaction_ < 0.05).

**Conclusion:**

Calcium intake was positively associated with aggressive prostate cancer, while vitamin D intake exhibited an inverse relationship. However, these associations varied by race/ethnicity and BMI. The findings from this study may help develop better prostate cancer prevention and management strategies.

**Electronic supplementary material:**

The online version of this article (doi:10.1186/s12885-017-3060-8) contains supplementary material, which is available to authorized users.

## Background

Prostate cancer (PCa) is the most common cancer among men in the U.S., and African American (AA) men have higher incidence and mortality rates compared to European American (EA) men and other racial/ethnic groups [1]. Nutrition and physical activity are key factors for cancer prevention [2], and several mechanistic roles have been proposed for calcium and vitamin D in PCa pathogenesis and progression [3, 4]. However, epidemiologic studies do not support findings from in vitro studies. Many epidemiologic studies have shown that dairy intake increases risk of overall PCa, aggressive PCa, and mortality [3, 5–9], while other studies found no association [10, 11].

Dairy products have two key nutrients, calcium and vitamin D, that may interact in PCa pathogenesis and progression, or may independently affect PCa. Several epidemiologic studies have shown that high calcium intake increases risk of overall PCa, advanced PCa, and PCa mortality [3, 5, 6, 11, 12]. In contrast, epidemiologic studies failed to link vitamin D intake with a reduced risk for PCa [5, 7]. Most epidemiologic studies have been conducted mainly in European descent populations, and only a few have explored the association of calcium and vitamin D intake with PCa in AAs [7, 13]. In one of these studies, Rowland et al. [13] found that high calcium intake in AA men increases PCa risk. Thus, it is necessary to further explore this relationship in this high risk population.

In the present study, we investigated whether calcium and vitamin D intake were associated with PCa diagnosis and aggressiveness in men from a multiethnic population from Chicago, IL and Washington, D.C., and if associations of calcium and vitamin D intake differed based on race/ethnicity and body mass index (BMI). For AAs living in high latitude environments, such as Chicago, where adequate ultraviolet radiation for cutaneous vitamin D synthesis is available only for a few months of year, vitamin D intake is a major source of vitamin D. We and others [14–17] have shown that vitamin D intake correlates strongly with serum vitamin D levels. We also evaluated whether the Institute of Medicine (IOM)-recommended dosage amount, Recommended Dietary Allowance (RDA), for calcium and vitamin D intake, were beneficial for PCa prevention. The IOM recommendations were developed for general populations, despite racial disparities in cutaneous vitamin D synthesis and vitamin D deficiency. Thus, we sought to determine if AAs who are at higher risk for PCa and vitamin D deficiency can benefit equally to EAs from following the recommendations.

## Methods

### Study participants

We recruited a total of 2,322 study participants for cross-sectional studies among controls and men who underwent PCa biopsy (1,381 AAs, 715 EAs, and 226 from other racial/ethnic backgrounds). PCa patients and controls ages 40 to 79 years old were recruited from six public and academic hospitals in Chicago, IL (Cook County Health and Hospital System, Northwestern Memorial Hospital, Jesse Brown Veterans Affairs Medical Center, University of Illinois Hospital and Health Science System, and University of Chicago Hospital) between 2009 and 2014 [18] and Washington, D.C. (Howard University Hospital) between 2000 and 2004 [19]. The patients underwent their first prostate biopsy due to an elevated or abnormal serum prostate specific antigen (PSA) level or an abnormal digital rectal examination. The patients were recruited before they underwent prostate biopsy. PCa diagnosis was histologically confirmed for all cases. Controls were patients who were recruited at urology clinics without history of PCa and healthy AA men who do not have history of PCa or other types of cancer were recruited at community health events. In our analysis, we excluded 665 study participants, due to missing dietary information (*n* = 395) and no prostate biopsy (*n* = 3) for individuals with elevated PSA and abnormal digital rectal exam results. Patients who had negative biopsy (*n* = 239) or who had history of other types of cancer (*n* = 28) were also excluded. After removing these individuals, a total of 1,657 men (699 PCa patients and 958 controls) were available for analysis. The Institutional Review Board of the University of Illinois at Chicago approved the research protocol.

At the time of recruitment, clinical research coordinators obtained information on calcium and vitamin D intake, dietary supplement use, age, height, weight, family history of PCa, education, alcohol and tobacco use, and marital status. Men at high risk for aggressive PCa were determined using the National Comprehensive Cancer Network (NCCN) risk stratification scheme, which has been used for predicting biochemical failure [20]. Following the NCCN guidelines, patients who were classified as having high risk PCa had a Gleason Score ≥4 + 4, PSA level ≥20.0 ng/mL, or clinical stage ≥ T3a,N0,M0﻿.

### Dietary assessment

Self-reported calcium and vitamin D intake were evaluated using the Block calcium and vitamin D screener, a food frequency questionnaire (FFQ) developed from the National Health and Nutrition Examination Survey (NHANES) 1999–2001 dietary recall data. The Block screener has been validated for use in the AA population [21]. The questionnaire includes 19 food items, 3 supplement questions, and items to adjust for food fortification practices. Participants were asked about the amount of consumption (serving size) and frequency of food consumption (never, 2–3 times per month, 1–2 times per week, 3–4 times per week, 5–6 times per week, or every day) in the past year. A research coordinator administered the FFQ at the time of recruitment. The completed FFQs were sent to NutritionQuest in Berkeley, CA where the proprietary software was used for analysis to calculate vitamin D and calcium intake. Dietary intake is from food items, while supplemental intake includes fortification and dietary supplement intake. Total intake combines dietary and supplemental intake. Vitamin D intake is reported in International Unit (IU, 1 IU = 0.025 μg).

### Statistical analysis

A student’s *t*-test or Mann-Whitney *U* test for continuous variables and *χ* ^2^ test for categorical variables were used to examine study participants’ characteristics. We investigated the associations of dietary, supplemental, and total calcium and vitamin D intake with PCa diagnosis using unconditional logistic regression analyses. Quartiles based on dietary and total calcium and vitamin D intake were used as independent variables. Because it was not possible to make categories using medians, tertiles or quartiles for supplemental calcium and vitamin D intake due to skewed distributions of supplemental intake, different categorization schemes were used (supplemental calcium intake 0, 1–199, and ≥200 mg/day and supplemental vitamin D intake 0, 1–399, and ≥400 IU/day) following Park et al. [7]. The final adjusted logistic regression model included age at diagnosis for PCa cases or age of recruitment for controls, family history of PCa (yes or no/unknown), race/ethnicity (AA, EA, Hispanic Americans, and others), BMI [weight (kg)/height (m)^2^], education (<high school/high school, associate/technical/bachelor degree, or master/PhD/professional degree), smoking (no, current smoker, former smoker), alcohol use (no, yes but quit, or currently use alcohol), and marital status (married/living like married or single/divorced/widowed). Age and BMI were modeled as continuous variables. In the models, we simultaneously adjusted for total calcium and total vitamin D intake as well as dietary and supplemental intake to evaluate if the associations with PCa were independent.

In stratified analyses of race and BMI, we used quartiles of calcium and vitamin D intake instead of tertiles to maximize the contrast between low and high intake groups, even though number of patients in each quartile was small. For analyses stratifying based on the participants’ BMI, we used the median BMI as the cut-off (<27.8 vs. ≥27.8 kg/m^2^). We tested linear trends by assigning study participants the median value of each quartile and treating it as a continuous variable. For the analysis of the association between dietary and supplement intake and PCa diagnosis, we used the recommendations set by the IOM as cut-off points in our analysis. The IOM set Estimated Average Requirement (EAR) for adults age between 19 and 70 for calcium intake as 800 mg/day and for vitamin D intake as 400 IU/day [22]. The IOM Recommended Dietary Allowance (RDA) for calcium is 1,000 mg/day and for vitamin D is 600 IU/day. We categorized total calcium intake (<800 mg/day, 800–1,000 mg/day, and ≥1,000 mg/day) and total vitamin D intake (<400 IU/day, 400–600 IU/day, and ≥600 IU/day) into three-level ordinal variables. We did not categorize dietary vitamin D and supplemental calcium intake in this way since few participants consumed the recommended amount. SPSS statistical software version 22.0 (IBM Corp., Armonk, NY) was used for analyses.

## Results

The PCa patients were older than controls (mean age of 63.8 and 58.9 in cases and controls respectively, *P* < 0.001) (Table 1). There were 888 AAs, 620 EAs, 111 Hispanic Americans, and 38 individuals from other racial/ethnic groups (Asian and Middle Eastern Americans). Overall, a small proportion of study participants consumed the RDA for calcium (26.3%) and vitamin D (19.1%), and median total calcium and vitamin D intake was considerably lower than the RDA. PCa patients had slightly higher total calcium intake, while controls had significantly higher supplemental vitamin D intake (*P* = 0.02). The distribution of supplemental calcium and vitamin D intake was skewed. Many study participants did not have supplemental calcium and vitamin D intake. In fact, 59.2% of participants had less than 100 mg/day of supplemental calcium intake. A large proportion of study participants (87.2%) had less than 200 mg/day of supplemental calcium intake, while 55.2% of participants consumed less than 100 IU/day of vitamin D. We observed significantly positive correlations between dietary calcium and vitamin D intake and between total calcium and vitamin D intake (*P* < 0.001), but there we many people who had low calcium intake while consuming a large amount of vitamin D.

**Table 1:** Study participants’ characteristics

	Cases (*n* = 699)	Controls (*n* = 958)	*P*
Age, mean (SD^1^)	63.3 (8.1)	58.9 (9.8)	<0.001
Body Mass Index, mean (SD)	28.2 (5.0)	29.2 (6.0)	<0.001
Race/Ethnicity			0.25
African Americans/African Descents	391 (55.9)	497 (51.9)	
European Americans	242 (34.6)	378 (39.5)	
Hispanic Americans	50 (7.2)	61 (6.4)	
Others	16 (2.3)	22 (2.3)	
Dietary Calcium Intake (mg/day), median (IQR^2^)	506.7 (281.0–826.4)	502.0 (286.5–800.6)	0.79
Supplemental Calcium Intake (mg/day), median (IQR)	0.0 (0.0–162.0)	22.7 (0.0–162.0)	0.23
Total Calcium Intake (mg/day), median (IQR)	645.2 (370.0–1049.5)	600.1 (367.1–1019.0)	0.59
Dietary Vitamin D Intake (IU/day)^3^, median (IQR)	75.6 (32.1–152.7)	68.7 (28.5–147.9)	0.17
Supplemental Vitamin D Intake (IU/day), median (IQR)	0.0 (0.0–400.0)	26.8 (0.0–400.0)	0.02
Total Vitamin D Intake (IU/day), median (IQR)	237.2 (57.2–504.5)	245.8 (66.8–515.9)	0.25
Dietary Supplement Use, n (%)	94 (16.8)	185 (21.7)	0.02
Education, n (%)			<0.001
< High School or High School	326 (51.3)	378 (41.0)	
Some College, 4 Years of College	188 (29.6)	316 (34.3)	
Master, PhD, and Professional Degree	122 (19.2)	227 (24.6)	
Income, n (%)			<0.001
< $30,000	286 (42.2)	296 (31.8)	
$30,000–60,000	159 (23.5)	239 (25.7)	
≥ $60,000	232 (34.3)	395 (42.5)	
Married or Living Like Married, n (%)	390 (56.0)	550 (57.8)	0.45
Smoking, n (%)			0.08
Current Smoker	122 (17.6)	158 (16.7)	
Yes, but Quit	267 (38.6)	321 (34.0)	
Alcohol Use, n (%)			0.02
Yes, but Quit	149 (21.3)	201 (21.2)	
Currently Drink	447 (64.0)	560 (58.9)	
Family History, n (%)	163 (23.3)	199 (12.5)	<0.001
PSA, median (IQR)	6.5 (7.7)	1.2 (1.7)	<0.001
Aggressive PCa			
Greater than High Risk PCa^4^, n (%)	157 (23.5)		
Gleason Score 4 + 3 or Greater, n (%)	155 (24.3)		
Gleason Score 4 + 4 or Greater, n (%)	91 (14.3)		

^1^Standard Deviation (SD)

^2^Interquartile Range (IQR)

^3^Vitamin D International Unit (IU, 1 IU = 0.025 μg)

^4^Based on National Comprehensive Cancer Network (NCCN) risk stratification for biochemical failure

AA and EA study participants had a similar total calcium intake, but they exhibited different dietary and supplemental calcium intake patterns (Additional file 1: Table S1). Other behavioral and sociodemographic characteristics that could contribute to PCa risk were also different. AA men had significantly higher dietary vitamin D intake than EA men (*P* < 0.001), but EA men tended to have higher supplemental and total vitamin D intake (*P* < 0.001) and were more likely to use dietary supplements (26.1% in EAs compared to14.9% in AAs, *P* < 0.001). EA participants were also more likely to be married (*P* < 0.00), have higher education (*P* < 0.001), and use alcohol regularly (*P* < 0.001). There were more current smokers among AA than EA participants (*P* < 0.001). In addition, AA men were more likely to be in the high NCCN risk group than EA men (29.1% in AA patients vs. 14.5% in EA patients, *P* < 0.001).

In the pooled analysis including all populations, calcium and vitamin D intake were not associated with overall PCa risk (*P* > 0.05), but high total and dietary calcium intake were significantly associated with high NCCN risk group and high grade PCa (Table 2). Compared to men in the lowest total calcium intake quartile, the men in the highest quartile were almost two times more likely to have high risk PCa (OR = 1.98, 95% C.I.: 1.01–3.91). The association of the highest quartile of total and dietary calcium intake with Gleason Score ≥4 + 3 was not significant, but we observed statistically significant linear trends (*P*_Trend_ =0.03 for total calcium and *P*_Trend_ =0.02 for dietary calcium intake). Total vitamin D intake showed an inverse association for high risk PCa (OR = 0.38, 95% C.I.: 0.18–0.79). The association was stronger after adjusting for potential confounders. Dietary vitamin D intake was significantly positively associated with high risk PCa in our crude model, but showed no association after adjusting for relevant variables, such as total calcium intake. Supplemental calcium and vitamin D intake were not independently associated with PCa. Because calcium and vitamin D intake were highly correlated and showed opposing directions of association, we investigated the interaction between calcium and vitamin D intake. This interaction was not statistically significant.

**Table 2:** Association of dietary and total calcium and vitamin D intake with prostate cancer

	Controls		Cases vs. Controls			NCCN High Risk vs. Controls			Gleason Score ≥4 + 3 vs. Controls	
			Unadjusted	Adjusted		Unadjusted	Adjusted		Unadjusted	Adjusted
	n (%)	n (%)	OR (95% C.I.)	OR (95% C.I.)	n (%)	OR (95% C.I.)	OR (95% C.I.)	n (%)	OR (95% C.I.)	OR (95% C.I.)
Dietary Calcium, mg/day										
Quartile 1 (<283.5)	236 (24.6)	178 (25.5)	1.00	1.00	35 (22.3)	1.00	1.00	30 (19.4)	1.00	1.00
Quartile 2 (283.5–504.4)	246 (25.7)	168 (24.0)	0.91 (0.69–1.19)	0.86 (0.63–1.18)	32 (20.4)	0.88 (0.53–1.46)	0.88 (0.48–1.61)	33 (21.3)	1.06 (0.62–1.79)	0.99 (0.55–1.79)
Quartile 3 (504.5–813.1)	247 (25.8)	168 (24.0)	0.90 (0.68–1.19)	0.93 (0.67–1.30)	35 (22.3)	0.96 (0.58–1.58)	1.37 (0.73–2.57)	38 (24.5)	1.21 (0.73–2.02)	1.47 (0.82–2.66)
Quartile 4 (>813.1)	229 (23.9)	185 (26.5)	1.07 (0.81–1.41)	0.98 (0.69–1.39)	55 (35.0)	1.62 (1.02–2.57)	1.43 (0.76–2.71)	54 (34.8)	1.86 (1.15–3.00)	1.80 (1.00–3.25)
* P* for Trend			0.49	0.92		0.04	0.07		0.03	**0.02**
Dietary Vitamin D, IU/day										
Quartile 1 (<29.9)	249 (26.0)	165 (23.6)	1.00	1.00	26 (16.6)	1.00	1.00	29 (18.7)	1.00	1.00
Quartile 2 (29.9–71.4)	244 (25.5)	170 (24.3)	1.05 (0.80–1.39)	0.98 (0.71–1.35)	41 (26.1)	1.61 (0.96–2.71)	1.24 (0.68–2.29)	37 (23.9)	1.30 (0.77–2.18)	1.03 (0.58–1.84)
Quartile 3 (71.5–148.3)	229 (23.9)	186 (26.6)	1.23 (0.93–1.62)	1.15 (0.81–1.63)	48 (30.6)	2.01 (1.21–3.34)	1.45 (0.75–2.80)	46 (29.7)	1.73 (1.05–2.84)	1.28 (0.69–2.37)
Quartile 4 (>148.3)	236 (24.6)	178 (25.5)	1.14 (0.86–1.50)	1.01 (0.67–1.50)	42 (26.8)	1.70 (1.01–2.86)	0.86 (0.40–1.86)	43 (27.7)	1.56 (0.95–2.59)	0.96 (0.48–1.93)
* P* for Trend			0.35	0.87		0.13	0.38		0.11	0.93
Supplemental Calcium, mg/day										
0	464 (48.4)	384 (54.9)	1.00	1.00	94 (59.9)	1.00	1.00	83 (53.5)	1.00	1.00
0–400	103 (10.8)	41 (5.9)	0.48 (0.33–0.71)	0.66 (0.2–1.03)	8 (5.1)	0.38 (0.18–0.81)	0.67 (0.28–1.59)	11 (7.1)	0.60 (0.31–1.16)	1.02 (0.48–2.14)
≥400	391 (40.8)	274 (39.2)	0.85 (0.69–1.04)	0.88 (0.69–1.13)	55 (35.0)	0.69 (0.49–0.99)	0.64 (0.40–1.01)	61 (39.4)	0.87 (0.61–1.25)	0.79 (0.52–1.22)
* P* for Trend			0.20	0.39		0.07	0.05		0.54	0.29
Total Calcium, mg/day										
Quartile 1 (<368.8)	240 (25.1)	174 (24.9)	1.00	1.00	34 (21.7)	1.00	1.00	30 (19.4)	1.00	1.00
Quartile 2 (368.8–616.0)	249 (26.0)	165 (23.6)	0.91 (0.69–1.21)	0.94 (0.67–1.31)	34 (21.7)	0.96 (0.58–1.60)	1.10 (0.59–2.02)	34 (21.9)	1.09 (0.65–1.84)	1.12 (0.62–2.05)
Quartile 3 (616.1–1033.3)	234 (24.4)	181 (25.9)	1.07 (0.81–1.41)	1.19 (0.84–1.68)	38 (24.2)	1.15 (0.70–1.88)	1.38 (0.72–2.63)	43 (27.7)	1.47 (0.89–2.42)	1.66 (0.91–3.04)
Quartile 4 (>1033.3)	235 (24.5)	179 (25.6)	1.05 (0.80–1.38)	1.06 (0.74–1.53)	51 (32.5)	1.53 (0.96–2.45)	**1.98 (1.01–3.91)**	48 (31.0)	1.63 (1.01–2.67)	1.83 (0.97–3.48)
* P* for Trend			0.49	0.68		0.04	**0.03**		0.03	**0.03**
Total Vitamin D, IU/day										
Quartile 1 (<63.5)	231 (24.1)	183 (26.2)	1.00	1.00	39 (24.8)	1.00	1.00	36 (23.2)	1.00	1.00
Quartile 2 (63.5–239.2)	244 (25.5)	170 (24.3)	0.88 (0.67–1.16)	0.86 (0.61–1.21)	39 (24.8)	0.95 (0.58–1.53)	0.85 (0.46–1.56)	38 (24.5)	1.00 (0.61–1.63)	0.91 (0.50–1.65)
Quartile 3 (239.3–510.2)	239 (24.9)	176 (25.2)	0.93 (0.71–1.22)	0.89 (0.63–1.25)	52 (33.1)	1.29 (0.82–2.03)	1.03 (0.57–1.89)	46 (29.7)	1.24 (0.77–1.98)	1.08 (0.60–1.93)
Quartile 4 (>510.2)	244 (25.5)	170 (24.3)	0.88 (0.67–1.16)	0.81 (0.56–1.16)	27 (17.2)	0.66 (0.39–1.11)	**0.38 (0.18–0.79)**	35 (22.6)	0.92 (0.56–1.52)	0.64 (0.33–1.23)
* P* for Trend			0.55	0.39		0.17	**0.01**		0.80	0.16

NOTE: Model adjusted for age, family history of PCa, race/ethnicity, BMI, education, smoking, alcohol use, and marital status as well as mutually adjustment for total calcium or vitamin D intake. In the adjusted model, dietary and supplementary calcium and vitamin D intake simultaneously adjusted for each other. For the analysis of dietary and supplementary calcium intake, total vitamin D intake was adjusted for, while we adjusted for total calcium intake for the analysis of dietary and supplemental vitamin D intake. Significant association is shown with bolded type

Because AA and EA patients exhibited very different demographic and dietary behavioral characteristics, we performed stratified analyses, and observed stronger associations in AAs than in EAs or in pooled dataset. In AAs, the highest quartile of total vitamin D intake was associated with 47% lower odds of PCa diagnosis (95% C.I.:0.30–0.94) (Table 3). Total vitamin D intake was strongly negatively associated with high risk PCa (OR_Quartile 1 vs. Quartile 4_ = 0.06, 95% C.I.: 0.02–0.21) and high grade PCa (OR_Quartile 1 vs. Quartile 4_ = 0.17, 95% C.I.: 0.06–0.54). High supplemental vitamin D intake was also associated with lower odds of high risk and high grade PCa (Additional file 2: Table S2). Both dietary and total calcium intake were associated with high risk and high grade PCa, and high total calcium intake increased odds of high risk PCa (OR_Quartile 1 vs. Quartile 4_ = 4.28, 95% C.I.: 1.70–10.80) and high grade (OR_Quartile 1 vs. Quartile 4_ = 3.42, 95% C.I.: 1.30–9.00). Conversely, we did not observe these relationships in EAs. Interestingly, although not statistically significant, the odds of high risk and high grade PCa for men who had high supplemental and total vitamin D were slightly increased. We tested interaction between calcium and vitamin D intake and race/ethnicity among AA and EA study participants, and the interaction between total vitamin D and race/ethnicity was significant for high risk PCa (*P*_Interaction_ = 0.007), but not overall PCa risk or high grade PCa. The interaction between supplemental vitamin D intake and race/ethnicity was also statistically significant (*P*_Interaction_ = 0.03) for high risk PCa.

**Table 3:** Association of total calcium and vitamin D intake with prostate cancer in African Americans and European Americans

	Controls		Cases vs. Controls			High Risk vs. Controls			Gleason Score ≥4 + 3 vs. Controls	
			Unadjusted	Adjusted		Unadjusted	Adjusted		Unadjusted	Adjusted
	n (%)	n (%)	OR (95% C.I.)	OR (95% C.I.)	n (%)	OR (95% C.I.)	OR (95% C.I.)	n (%)	OR (95% C.I.)	OR (95% C.I.)
Total Calcium, mg/day										
African Americans										
Quartile 1 (<368.8)	143 (28.8)	111 (28.4)	1.00	1.00	25 (23.8)	1.00	1.00	18 (21.7)	1.00	1.00
Quartile 2 (368.8–616.0)	113 (22.7)	90 (23.0)	1.03 (0.71–1.49)	0.94 (0.61–1.58)	21 (20.0)	1.06 (0.57–2.00)	1.39 (0.63–3.09)	17 (20.5)	1.20 (0.59–2.42)	1.47 (0.62–3.51)
Quartile 3 (616.1–1033.3)	123 (24.7)	91 (23.3)	0.95 (0.66–1.38)	1.20 (0.73–1.97)	24 (22.9)	1.12 (0.61–2.05)	1.88 (0.81–4.37)	23 (27.7)	1.49 (0.77–2.88)	2.32 (0.98–5.51)
Quartile 4 (>1033.3)	118 (23.7)	99 (25.3)	1.08 (0.75–1.56)	1.33 (0.78–2.26)	35 (33.3)	1.70 (0.96–3.00)	**4.28 (1.70–10.80)**	25 (30.1)	1.68 (0.88–3.23)	**3.42 (1.30–9.00)**
* P* for Trend			0.73	0.28		0.05	**0.001**		0.10	**0.01**
European Americans										
Quartile 1 (<368.8)	80 (21.2)	54 (22.3)	1.00	1.00	5 (14.3)	1.00	1.00	9 (15.5)	1.00	1.00
Quartile 2 (368.8–616.0)	115 (30.4)	60 (24.8)	0.77 (0.49–1.23)	0.84 (0.50–1.40)	10 (28.6)	1.39 (0.46–4.23)	1.66 (0.50–5.52)	15 (25.9)	1.16 (0.48–2.78)	1.27 (0.50–3.22)
Quartile 3 (616.1–1033.3)	87 (23.0)	72 (29.8)	1.23 (0.77–1.95)	1.19 (0.70–2.03)	8 (22.9)	1.47 (0.46–4.68)	1.63 (0.43–6.18)	16 (27.6)	1.64 (0.68–3.91)	1.05 (0.41–2.68)
Quartile 4 (>1033.3)	96 (25.4)	56 (23.1)	0.86 (0.54–1.39)	0.81 (0.46–1.43)	12 (34.3)	2.00 (0.68–5.92)	1.65 (0.44–6.16)	18 (31.0)	1.67 (0.71–3.91)	1.23 (0.48–3.11)
* P* for Trend			0.98	0.54		0.20	0.88		0.18	0.54
*P* for Interaction (Total Calcium Intake x Race)				0.70			0.99			0.99
Total Vitamin D, IU/day										
African Americans										
Quartile 1 (<63.5)	120 (24.1)	165 (23.6)	1.00	1.00	29 (27.6)	1.00	1.00	23 (27.7)	1.00	1.00
Quartile 2 (63.5–239.2)	139 (28.0)	170 (24.3)	0.87 (0.61–1.25)	0.83 (0.51–1.34)	29 (27.6)	0.86 (0.49–1.53)	0.57 (0.26–1.24)	22 (26.5)	0.83 (0.44–1.56)	0.54 (0.24–1.25)
Quartile 3 (239.3–510.2)	135 (27.2)	186 (26.6)	0.80 (0.56–1.15)	0.74 (0.45–1.21)	37 (35.2)	1.13 (0.66–1.96)	0.58 (0.26–1.27)	28 (33.7)	1.08 (0.59–1.98)	0.69 (0.31–1.55)
Quartile 4 (>510.2)	103 (20.7)	178 (25.5)	0.71 (0.48–1.07)	**0.53 (0.30–0.94)**	10 (9.5)	0.40 (0.19–0.86)	**0.06 (0.02–0.21)**	10 (12.0)	0.51 (0.23–1.11)	**0.17 (0.06–0.54)**
* P* for Trend			0.11	**0.03**		0.05	**<0.001**		0.18	**0.008**
European Americans										
Quartile 1 (<63.5)	88 (23.3)	56 (23.1)	1.00	1.00	9 (25.7)	1.00	1.00	12 (20.7)	1.00	1.00
Quartile 2 (63.5–239.2)	81 (21.4)	44 (18.2)	0.85 (0.52–1.40)	0.82 (0.47–1.44)	3 (8.6)	0.36 (0.10–1.38)	0.24 (0.05–1.11)	11 (19.0)	1.00 (0.42–2.38)	0.72 (0.27–1.93)
Quartile 3 (239.3–510.2)	89 (23.5)	57 (23.6)	1.01 (0.63–1.61)	1.09 (0.63–1.87)	10 (28.6)	1.10 (0.43–2.83)	1.08 (0.34–3.44)	14 (24.1)	1.15 (0.51–2.63)	1.05 (0.41–2.68)
Quartile 4 (>510.2)	120 (31.7)	85 (35.1)	1.11 (0.72–1.72)	1.25 (0.74–2.12)	13 (37.1)	1.06 (0.43–2.59)	1.32 (0.40–4.33)	21 (36.2)	1.28 (0.60–2.75)	1.23 (0.48–3.11)
* P* for Trend			0.37	0.14		0.39	0.15		0.44	0.36
*P* for Interaction (Total Vitamin D Intake x Race)				0.23			**0.007**			0.14

NOTE: Model adjusted for age, family history of PCa, BMI, education, smoking, alcohol use, and marital status as well as mutually adjustment for total calcium or vitamin D intake. Significant association is shown with bolded type

Next, we investigated if BMI modified the associations between calcium and vitamin D intake and PCa (Table 4, Additional file 3: Table S3). We observed stronger relationships between calcium and vitamin D intake and PCa in leaner men (BMI <27.8 kg/m^2^) compared to men with higher BMI (≥27.8 kg/m^2^). High total and dietary calcium intake increased the odds of high risk and high grade PCa in both groups, but the associations were significant only in the leaner group after adjustment. In the leaner group, high total calcium increased odds of diagnosis with high risk PCa (OR_Quartile 1 vs. Quartile 4_ = 1.25, 95% C.I.: 1.25–9.42) and high grade PCa (OR_Quartile 1 vs. Quartile 4_ = 2.69, 95% C.I.: 1.02–7.11). Total and supplemental vitamin D intake showed strong inverse associations in the leaner group, but such effect was not observed in the high BMI group. In leaner men, high total vitamin D intake reduced odds of PCa diagnosis (OR_Quartile 1 vs. Quartile 4_ = 0.57, 95% C.I.: 0.33–0.97), high risk PCa (OR_Quartile 1 vs. Quartile 4_ = 0.09, 95% C.I.: 0.02–0.35), and high grade PCa (OR_Quartile 1 vs. Quartile 4_ = 0.20, 95% C.I.: 0.07–70.60). The interaction between total calcium intake and BMI for high risk PCa was significant (*P*_Interaction_ = 0.05), and the interaction between total vitamin D intake and BMI on high risk and high grade PCa was also statistically significant (*P*_Interaction_ = 0.02 and 0.04 for high risk and high grade PCa respectively). Supplemental calcium intake exhibited a negative association with PCa in the lower BMI group, but the association was not significant in the adjusted models. High supplemental calcium intake, on the other hand, was associated with high risk PCa in the high BMI group. The interaction was significant (*P*_Interaction_ = 0.007).

**Table 4:** Association of total calcium and vitamin D intake with prostate cancer in stratified analysis based on body mass index

	Controls		Cases vs. Controls			High Risk vs. Controls			Gleason Score ≥4 + 3 vs. Controls	
			Unadjusted	Adjusted		Unadjusted	Adjusted		Unadjusted	Adjusted
	n (%)	n (%)	OR (95% C.I.)	OR (95% C.I.)	n (%)	OR (95% C.I.)	OR (95% C.I.)	n (%)	OR (95% C.I.)	OR (95% C.I.)
Total Calcium, mg/day										
BMI < Median (27.8)										
Quartile 1 (<368.8)	93 (21.5)	90 (25.9)	1.00	1.00	15 (18.8)	1.00	1.00	13 (18.1)	1.00	1.00
Quartile 2 (368.8–616.0)	124 (28.7)	73 (21.6)	0.63 (0.42–0.94)	0.76 (0.47–1.24)	21 (26.3)	1.05 (0.51–2.15)	1.82 (0.74–4.43)	19 (26.4)	1.10 (0.52–2.33)	1.46 (0.60–3.54)
Quartile 3 (616.1–1033.3)	110 (25.5)	92 (26.4)	0.86 (0.58–1.29)	1.06 (0.64–1.75)	18 (22.5)	1.02 (0.49–2.12)	1.25 (0.48–3.25)	19 (26.4)	1.24 (0.58–2.64)	1.57 (0.64–3.90)
Quartile 4 (>1033.3)	105 (24.3)	91 (26.1)	0.90 (0.60–1.34)	1.13 (0.67–1.92)	26 (32.5)	1.54 (0.77–3.07)	**3.43 (1.25–9.42)**	21 (29.2)	1.43 (0.68–3.02)	**2.69 (1.02–7.11)**
* P* for Trend			0.80	0.36		0.17	**0.02**		0.30	**0.045**
BMI ≥ Median (27.8)										
Quartile 1 (<368.8)	127 (27.2)	80 (24.0)	1.00	1.00	17 (24.3)	1.00	1.00	15 (19.5)	1.00	1.00
Quartile 2 (368.8–616.0)	109 (23.3)	85 (25.4)	1.24 (0.83–1.84)	1.18 (0.74–1.86)	11 (15.7)	0.75 (0.34–1.68)	0.79 (0.32–1.96)	13 (16.9)	1.01 (0.46–2.22)	1.02 (0.44–2.41)
Quartile 3 (616.1–1033.3)	112 (24.0)	87 (26.0)	1.23 (0.83–1.83)	1.38 (0.85–2.23)	19 (27.1)	1.28 (0.63–2.56)	1.77 (0.71–4.42)	23 (29.9)	1.74 (0.87–3.50)	1.96 (0.85–4.53)
Quartile 4 (>1033.3)	119 (25.5)	82 (24.6)	1.09 (0.74–1.63)	0.99 (0.60–1.67)	23 (32.9)	1.44 (0.74–2.84)	1.48 (0.56–3.91)	26 (33.8)	1.85 (0.93–3.66)	1.47 (0.61–3.55)
* P* for Trend			0.81	0.78		0.14	0.32		0.04	0.32
*P* for Interaction (Total Calcium Intake x BMI)				0.35			0.05			0.40
Total Vitamin D, IU/day										
BMI < Median (27.8)										
Quartile 1 (<63.5)	87 (20.1)	89 (25.6)	1.00	1.00	18 (22.5)	1.00	1.00	18 (25.0)	1.00	1.00
Quartile 2 (63.5–239.2)	109 (25.2)	90 (25.9)	0.81 (0.54–1.21)	0.85 (0.52–1.41)	20 (25.0)	0.89 (0.44–1.78)	0.86 (0.35–2.07)	18 (25.0)	0.80 (0.39–1.63)	0.78 (0.33–1.84)
Quartile 3 (239.3–510.2)	106 (24.5)	95 (27.3)	0.88 (0.58–1.31)	0.89 (0.54–1.48)	35 (43.8)	1.60 (0.85–3.01)	1.28 (0.54–3.03)	27 (37.5)	1.23 (0.64–2.38)	1.06 (0.46–2.43)
Quartile 4 (>510.2)	130 (30.1)	74 (21.3)	0.56 (0.37–0.84)	**0.57 (0.33–0.97)**	7 (8.8)	0.26 (0.10–0.65)	**0.09 (0.02–0.35)**	9 (12.5)	0.34 (0.14–0.78)	**0.20 (0.07–0.60)**
* P* for Trend			0.009	**0.03**		0.009	**0.001**		0.02	**0.004**
BMI ≥ Median (27.8)										
Total Vitamin D, IU/day										
Quartile 1 (<63.5)	125 (26.8)	88 (26.3)	1.00	1.00	18 (25.7)	1.00	1.00	15 (19.5)	1.00	1.00
Quartile 2 (63.5–239.2)	115 (24.6)	80 (24.0)	0.99 (0.67–1.47)	0.87 (0.54–1.40)	19 (27.1)	1.15 (0.57–2.29)	0.82 (0.33–2.02)	20 (26.0)	1.45 (0.71–2.97)	1.02 (0.43–2.40)
Quartile 3 (239.3–510.2)	117 (25.1)	72 (21.6)	0.87 (0.59–1.31)	0.88 (0.54–1.42)	14 (20.0)	0.83 (0.40–1.75)	0.73 (0.29–1.84)	16 (20.8)	1.14 (0.54–2.41)	1.07 (0.45–2.53)
Quartile 4 (>510.2)	110 (23.6)	94 (28.1)	1.21 (0.83–1.79)	1.16 (0.71–1.90)	19 (27.1)	1.20 (0.60–2.40)	0.81 (0.31–2.12)	26 (33.8)	1.97 (0.99–3.91)	1.37 (0.57–3.28)
* P* for Trend			0.30	0.34		0.75	0.78		0.08	0.38
*P* for Interaction (Total Vitamin D Intake x BMI)				0.12			**0.02**			**0.04**

NOTE: Model adjusted for age, family history of PCa, race/ethnicity, education, smoking, alcohol use, and marital status as well as mutually adjustment for total calcium or vitamin D intake. Significant association is shown with bolded type

We further stratified the study participants based on race/ethnicity to investigate if BMI differentially modified the associations between vitamin D and calcium intake and PCa in AAs and EAs. In AAs, total calcium and vitamin D intake were significantly associated with PCa in the leaner group, while the high BMI group showed no association (Additional file 4: Table S4). On the other hand, EAs in the high BMI group showed a statistically significant positive linear trend of increasing PCa risk with total vitamin D intake (*P*_Trend_ = 0.03). Interactions of BMI with total calcium and vitamin D intake were not significant in both races.

Finally, we evaluated the associations between the IOM daily intake recommendations for calcium and vitamin D with PCa diagnosis (Table 5). A larger proportion of PCa cases consumed more than the EAR (≥800 mg/day) of total calcium intake than controls (39.7% and 35.0% respectively). Men who consumed total calcium intake above the EAR had increased odds of overall PCa, and men who consumed total calcium intake between 800 and 1000 mg/day had significantly increased odds of overall PCa (OR = 1.61, 95% C.I.: 1.12–2.29). Dietary and total calcium intake of more than the EAR was also significantly increased odds of high grade PCa (*P*_Trend_ < 0.05). Conversely, a larger proportion of controls consumed more than the RDA (≥600 IU/day) of vitamin D intake compared to PCa patients (21.2% and 16.5% respectively). Total vitamin D intake showed a statistically non-significant inverse association, and total vitamin D intake of more than the RDA showed a trend for significantly reduced PCa risk (OR = 0.74, 95% C.I.: 0.54–1.01). Having more than the RDA for total vitamin D also significantly reduced odds of high risk PCa (OR = 0.44, 95% C.I.: 0.23–0.84). In addition, men who take more than the RDA of supplemental vitamin D had significantly reduced odds of overall PCa as well as high risk and high grade PCa.

**Table 5:** Institute of medicine calcium and vitamin D dietary reference intakes and prostate cancer

	Controls		Cases vs. Controls			NCCN High Risk vs. Controls			Gleason Score ≥4 + 3 vs. Controls	
			Unadjusted	Adjusted		Unadjusted	Adjusted		Unadjusted	Adjusted
	n (%)	n (%)	OR (95% C.I.)	OR (95% C.I.)	n (%)	OR (95% C.I.)	OR (95% C.I.)	n (%)	OR (95% C.I.)	OR (95% C.I.)
Dietary Calcium, mg/day										
<800	719 (75.1)	508 (72.7)	1.00	1.00	101 (64.3)	1.00	1.00	99 (63.9)	1.00	1.00
800–1000	93 (9.7)	73 (10.4)	1.11 (0.80–1.54)	1.04 (0.72–1.51)	20 (12.7)	1.53 (0.91–2.59)	1.18 (0.62–2.26)	23 (14.8)	1.80 (1.09–2.97)	**1.82 (1.03–3.20**)
≥1000	146 (15.2)	118 (16.9)	1.14 (0.88–1.50)	1.13 (0.83–1.55)	36 (22.9)	1.76 (1.15–2.67)	1.50 (0.88–1.55)	33 (21.3)	1.64 (1.07–2.53)	1.60 (0.97–2.65)
* P*			0.55	0.74		0.02	0.32		0.009	**0.04**
Dietary Vitamin D, IU/day										
<400	940 (98.1)	683 (97.7)	1.00	1.00	148 (94.3)	1.00	1.00	149 (96.1)	1.00	1.00
≥400	18 (1.9)	16 (2.3)	1.22 (0.62–2.42)	1.20 (0.53–2.74)	9 (5.7)	3.18 (1.40–7.20)	2.56 (0.84–7.85)	6 (3.9)	2.10 (0.82–5.38)	2.22 (0.71–6.96)
* P*			0.56	0.66		0.006	0.10		0.12	0.17
Supplemental Calcium, mg/day										
<800	908 (94.8)	656 (93.8)	1.00	1.00	145 (92.4)	1.00	1.00	144 (92.9)	1.00	1.00
≥800	50 (5.2)	43 (6.2)	1.19 (0.78–1.81)	1.07 (0.66–1.74)	12 (7.6)	1.50 (0.78–2.89)	1.90 (0.81–4.47)	11 (7.1)	1.39 (0.71–2.73)	1.46 (0.67–3.21)
* P*			0.42	0.79		0.22	0.14		0.34	0.35
Supplemental Vitamin D, IU/day										
<400	567 (59.2)	425 (60.8)	1.00	1.00	102 (65.0)	1.00	1.00	94 (60.6)	1.00	1.00
400–600	246 (25.7)	199 (28.5)	1.08 (0.86–1.35)	1.05 (0.81–1.36)	46 (29.3)	1.04 (0.71–1.52)	0.94 (0.59–1.49)	47 (30.3)	1.15 (0.79–1.69)	1.06 (0.69–1.64)
≥600	145 (15.1)	75 (10.7)	0.69 (0.51–0.94)	**0.70 (0.50–0.98)**	9 (5.7)	0.35 (0.17–0.70)	**0.35 (0.16–0.77)**	14 (9.0)	0.58 (0.32–1.05)	**0.47 (0.25–0.90)**
* P*			0.03	0.08		0.01	**0.03**		0.11	**0.047**
Total Calcium, mg/day										
<800	623 (65.0)	422 (60.4)	1.00	1.00	85 (54.1)	1.00	1.00	81 (52.3)	1.00	1.00
800–1000	85 (8.9)	92 (13.2)	1.60 (1.16–2.20)	**1.61 (1.12–2.29)**	19 (12.1)	1.64 (0.95–2.83)	1.55 (0.82–2.95)	24 (15.5)	2.17 (1.31–3.61)	**2.26 (1.30–3.95)**
≥1000	250 (26.1)	185 (26.5)	1.01 (0.87–1.37)	1.05 (0.80–1.38)	53 (33.8)	1.55 (1.07–2.26)	1.48 (0.91–2.40)	50 (32.3)	1.54 (1.05–2.25)	1.54 (0.98–2.43)
* P*			0.02	**0.03**		0.02	0.18		0.004	**0.009**
Total Vitamin D, IU/day										
<400	545 (56.9)	406 (58.1)	1.00	1.00	93 (59.2)	1.00	1.00	87 (56.1)	1.00	1.00
400–600	210 (21.9)	178 (25.5)	1.14 (0.90–1.44)	1.11 (0.85–1.46)	46 (29.3)	1.28 (0.87–1.89)	1.14 (0.71–1.83)	42 (27.1)	1.25 (0.84–1.87)	1.24 (0.80–1.95)
≥600	203 (21.2)	115 (16.5)	0.76 (0.59–0.99)	0.74 (0.54–1.01)	18 (11.5)	0.52 (0.31–0.88)	**0.44 (0.23–0.84)**	26 (16.8)	0.80 (0.50–1.28)	0.60 (0.35–1.05)
* P*			0.03	0.06		0.009	**0.02**		0.25	0.06

NOTE: Model adjusted for age, family history of PCa, race/ethnicity, BMI, education, smoking, alcohol use, and marital status, as well as mutually adjustment for total calcium or vitamin D intake. For the analysis of dietary and supplemental calcium intake, total vitamin D intake was adjusted for, while we adjusted for total calcium intake for the analysis of dietary and supplementary vitamin D intake. In addition, dietary and supplement intake was mutually adjusted. The IOM EAR for calcium intake is 800 mg/day and for vitamin D intake is 400 IU/day. The RDA for calcium is 1,000 mg/day and for vitamin D is 600 IU/day. Significant association is shown with bolded type

**Fig. 1:**
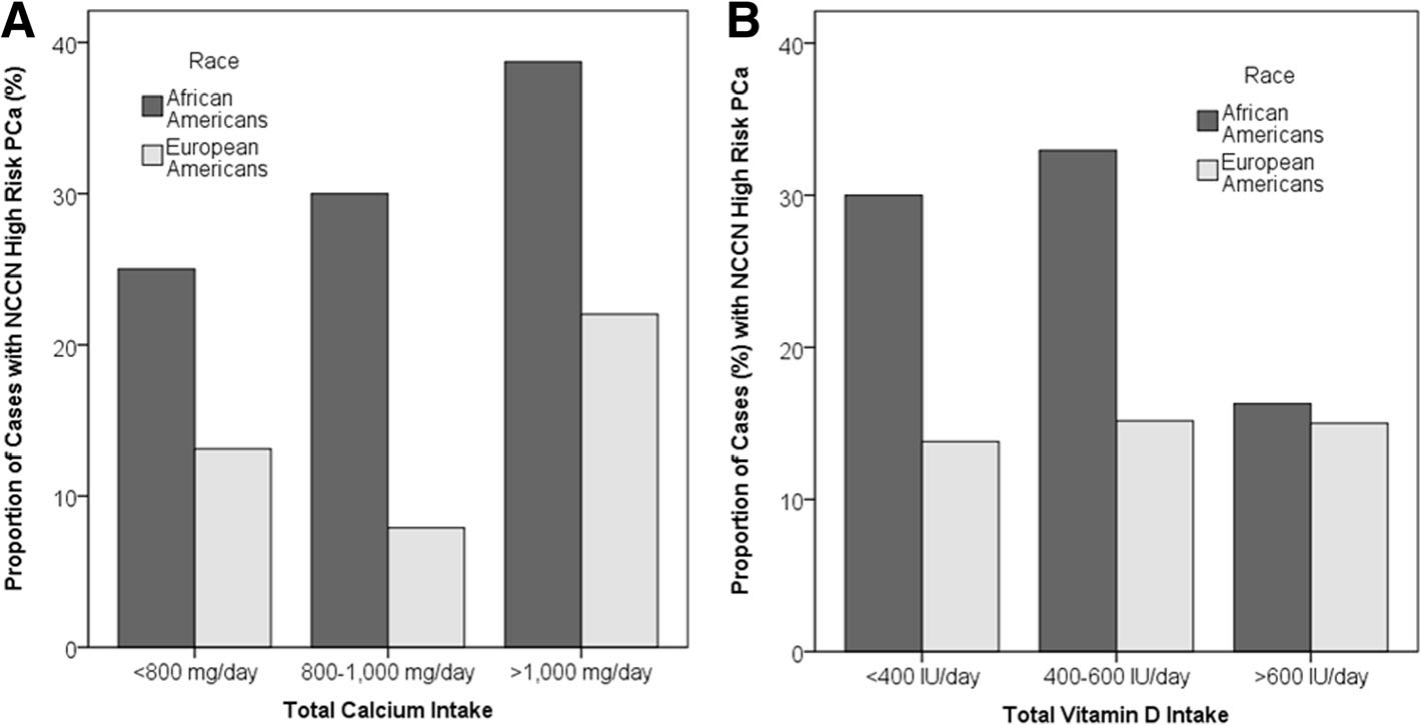
Proportion of cases with NCCN high risk PCa (%) and total calcium (**a**) and vitamin D (**b**) intake stratified based on raceᅟ

When the study participants were stratified based on race, total calcium intake above the EAR increased odds of overall PCa and aggressive PCa in both AAs and EAs. Total calcium intake between 800 and 1000 mg/day was significantly associated with increased odds of overall PCa in EAs and high grade PCa in AAs (Additional file 5: Table S5). Vitamin D intake above the RDA was negatively associated with overall PCa risk, and high risk and high grade PCa in AAs, but not in EAs. In AAs, the proportion of cases diagnosed with NCCN high risk PCa increases with total calcium intake in AAs (Fig. 1a), while the proportion of cases diagnosed with NCCN high risk PCa was lower among AA patients with vitamin D intake of more than the RDA and it was similar to that of EA patients (Fig. 1b). We did not observe such patterns in EAs, however.

## Discussion

In previous epidemiologic studies conducted mainly in European descent populations, the relationships between calcium and vitamin D intake and PCa were unclear. In this study of PCa in a multiethnic population, we evaluated this relationship, and found positive associations with high calcium intake and inverse relationships with high vitamin D intake. Men with high calcium intake were more likely to be diagnosed with NCCN high risk and high grade PCa, while men with high vitamin D intake were less likely to be diagnosed with high risk PCa. We also observed that the associations between calcium and vitamin D intake and PCa were modified by race/ethnicity and BMI. The relationships between high calcium and vitamin D intake with PCa were stronger in AAs and men with low BMI. Vitamin D intake above the RDA was associated with reduced odds of PCa in AAs, while calcium intake above the EAR was related to increased odds of PCa in AAs as well as EAs.

Recently, the World Cancer Research Fund International concluded that evidence linking high calcium intake to PCa risk is limited [23], but the results of the current work are consistent with many other studies that demonstrated that high calcium intake increases risk of PCa [3, 5, 6, 13] as well as reports showing associations between high serum calcium levels and increasing PCa risk and risk of fatal PCa [24, 25]. Epidemiologic evidence also does not provide definitive support for an inverse association between vitamin D and PCa [26–28], and other studies that explored the relationship between vitamin D intake and PCa did not find significant associations [5, 7]. However, previously, we demonstrated that serum vitamin D levels were associated with prostate biopsy outcome and adverse pathology after undergoing radical prostatectomy in our study group [18, 29], and this is the first study to demonstrate significant association between vitamin D intake and PCa. Compared to other work, our study has the advantage of including a large number of AA participants and men living in a low ultraviolet radiation environment who were vitamin D deficient [14, 15].

The primary hypothesis regarding how calcium may increase PCa risk is related to the interaction of calcium and 1,25-dihydroxyvitamin D3 [1,25(OH)_2_D], the active form of vitamin D [11]. Production of 1,25(OH)_2_D in the kidney is regulated by parathyroid hormone (PTH) in response to low serum calcium concentrations, and high serum calcium concentrations lowers 1,25(OH)_2_D concentrations [30]. Because 1,25(OH)_2_D has been shown to inhibit growth of PCa cells [31], suppression of its production at the cellular level by high levels of calcium would likely abrogate anti-carcinogenic effects. Alternatively, high consumption of dairy products, a major source of dietary calcium in the U.S., increases concentration of insulin-like-growth factor I (IGF-I) in serum [32]. IGF-I is involved in cell proliferation, differentiation, and apoptosis, and high circulating IGF-I levels may increase PCa risk [33].

We observed significant associations in AAs for calcium and vitamin D intake, but not in EAs, and significant interactions between race/ethnicity and supplemental calcium and vitamin D intake were observed. Non-significant associations in EAs could be due to small size for EAs. However, uncontrolled factors and residual confounding or measurement method may have affected our analyses. While our study participants had similar calcium and vitamin D intake as other studies conducted in the U.S. [16, 34], including a study among veterans in Chicago [35], other studies report higher calcium intake [13, 36, 37]. The AA and EA study participants also exhibited different socioeconomic and behavioral characteristics. Low-income individuals are less likely to meet dietary guidelines, and adherence to dietary recommendations is lower in AAs [38]. Studies also show that AAs prefer different foods than EAs [39], and dairy consumption is lower in AAs than other ethnic groups [40, 41]. Along with low dietary supplement usage, these factors partly contributed to lower calcium and vitamin D intake in our study participants. PTH concentrations in serum are also different between AAs and EAs. AAs generally have higher PTH concentrations, and PTH concentrations vary with BMI categories [35, 42]. In addition, there are differences in genetic variations that affect calcium and vitamin D metabolism and signaling [13, 14].

We showed that BMI potentially modifies associations between calcium and vitamin D intake and PCa. Modifying effects of BMI on calcium and PCa have been previously reported. Singapore Chinese men with BMI < 22.9 kg/m^2^ (median) who had high calcium consumption had a significantly increased PCa risk, but no such association was observed in men with BMI ≥ 22.9 kg/m^2^ [43]. Although obesity is recognized as an important risk factor for PCa [23] and BMI predicts serum vitamin D concentrations [15, 17], modifying effects of BMI on the relationship between vitamin D and PCa have not been well explored. In our study, high vitamin D consumption had beneficial effects only in the leaner group. Because vitamin D is fat soluble and stored in adipose tissue, high BMI individuals may have reduced levels of bioavailable vitamin D [44], illustrating the importance of weight management in PCa prevention.

There are several limitations in this study. First, recall bias can significantly affect associations between dietary intake and disease outcomes in a case-control study [45]. In order to avoid recall bias, we recruited patients before they underwent prostate biopsy, so at the time of recruitment, the patients did not know whether they had PCa or not. Secondly, we used data from cross-sectional studies, so the causal relationship between dietary intake and PCa could not be assessed. Third, the uncontrolled factors that were not captured in this work may also have affected the association between calcium and vitamin D intake and PCa. Behavioral and socioeconomic factors that potentially affect PCa diagnosis were also associated with dietary intake, and results from unadjusted model were different from fully-adjusted models. Additionally adjusting for uncontrolled factors may attenuate the associations. For example, many patients were diagnosed with PCa after undergoing prostate biopsy due to elevated PSA levels. Health conscious men who have high calcium and vitamin D intake may be more likely to have regular PSA testing. These PSA screened patients usually have low risk and low grade PCa. In our study, however, high calcium intake was associated with high risk PCa, and high vitamin D consumption reduced odds of high risk and high grade PCa. In addition, although this study is one of the largest studies aiming to understand the role of calcium and vitamin D intake in AAs, the sample size of this study may have been insufficient, especially for high risk and high grade PCa patients and study participants who consumed more than recommended amount of calcium and vitamin D. As a result, we may have observed different associations between unadjusted and adjusted models and spuriously inflated associations in our stratified analysis. Moreover, other minority groups were underrepresented in our study. Results from this study may not be generalizable to other racial/ethnic groups that have very different dietary patterns from our study populations. The effect of calcium and vitamin D intake should be further investigated including more study participants from diverse racial/ethnic backgrounds. Finally, we evaluated association of dietary intake rather than serum vitamin D levels because vitamin D intake is strongly correlated with serum vitamin D levels [14–17]. The relationship between serum vitamin D concentrations and PCa will be explored further in our future studies.

Calcium and vitamin D are important nutrients, and they may have preventive effects against many health conditions [46]. Although toxicity from high vitamin D supplementation may be low [47], high calcium intake is associated with increased PCa risk as well as risk of cardiovascular disease [48] and kidney stones [49]. High calcium consumption might be harmful and for PCa prevention, high dose calcium supplementation and fortification should be avoided, especially among AA men. The U.S. Preventive Service Task Force (USPSTF) and the U.S. National Institute of Health (NIH) concluded that evidence is currently insufficient to recommend vitamin D supplementation for prevention of cancer for general population [50, 51]. For AAs, however, vitamin D intake above the IOM RDA through vitamin D supplementation without calcium might be beneficial for prevention of aggressive PCa. A large majority of AAs in our study did not have adequate amount of vitamin D intake and more than half of them were vitamin D deficient [14, 15]. Vitamin D supplementation trials have shown that vitamin D supplementation improves PCa clinical characteristics by reducing number of positive cores at repeat biopsy in active surveillance patients and post radical prostatectomy PSA levels [52, 53].

## Conclusions

In summary, we showed that high calcium intake is associated with increased risk of aggressive PCa, while high vitamin D intake was inversely associated. We observed stronger effect estimates for calcium and vitamin D intake on PCa in AAs and men with low BMI. The findings from this study may help develop better PCa prevention and management plans. While a large scale trial among AAs who are at higher risk for PCa is necessary, higher vitamin D intake above the IOM RDA using supplementation or fortification and avoidance of high calcium intake, may reduce the rates of aggressive PCa diagnosis in AAs.

## Additional files

Additional file 1: Table S1. African American and European American Study Subjects’ Characteristics. (PDF 389 kb)
Additional file 2: Table S2. Association of Dietary Calcium and Vitamin D Intake with Prostate Cancer in African Americans and European Americans. (PDF 278 kb)
Additional file 3: Table S3. Association of Dietary Calcium and Vitamin D Intake with Prostate Cancer in Stratified Analysis Based on Body Mass Index. (PDF 279 kb)
Additional file 4: Table S4. Association of Total Calcium and Vitamin D Intake with Prostate Cancer in Stratified Analysis Based on Body Mass Index in African Americans and European Americans. (PDF 263 kb)
Additional file 5: Table S5. Institute of Medicine (IOM) Calcium and Vitamin D Dietary Reference Intakes and Prostate Cancer Stratified Based on Race. (PDF 262 kb)

## Abbreviations

1,25(OH)D: 1,25-dihydroxyvitamin D3
AA: African Americans
BMI: Body mass index
EA: European Americans
EAR: Estimated average requirement
FFQ: Food frequency questionnaire
IGF-I: Insulin-like-Growth Factor I
IOM: Institute of medicine
IU: International unit
NCCN: National Comprehensive Cancer Network
NHANES: National Health and Nutrition Examination Survey
NIH: National Institute of Health
PCa: Prostate cancer
PSA: Prostate specific antigen
PTH: Parathyroid hormone
RDA: Recommended dietary allowance
USPSTF: U.S. Preventive Service Task Force
