# Supplementary material for: Race and BMI modify associations of calcium and vitamin D intake with prostate cancer
Source: BMC Cancer. 2017 Jan 19;17:64. doi: 10.1186/s12885-017-3060-8 (PMC5248493; doi:10.1186/s12885-017-3060-8)
Supplement: Additional file 1: Table S1. — African American and European American Study Subjects’ Characteristics. (PDF 389 kb) [file 12885_2017_3060_MOESM1_ESM.pdf]

Online Supplemental Material

Supplemental Table 1 African American and European American Study Subjects' Characteristics

	African Americans	European Americans	*P*
Age, mean (SD[1])	60.3 (10.1)	61.5 (8.1)	0.01
Body Mass Index, mean (SD)	28.9 (5.7)	28.6 (5.5)	0.38
Dietary Calcium Intake (mg/day), median (IQR[2])	497.5 (269.9-833.7))	485.6 (300.7-746.4))	0.05
Supplemental Calcium Intake (mg/day), median (IQR)	5.4 (0.0-162.0)	56.0 (0.0-162.2)	0.02
Total Calcium Intake (mg/day), median (IQR)	596.2 (328.7-1010.1)	617.8 (393.7-1016.7)	0.52
Dietary Vitamin D Intake (IU/day), median (IQR)	75.9 (33.4-159.3)	61.2 (24.6-136.8)	<0.001
Supplemental Vitamin D Intake (IU/day), median (IQR)	0.0 (0.0-400.0)	350.0 (0.0-400.0)	<0.001
Total Vitamin D Intake (IU/day, median (IQR)	186.5 (59.4-463.4)	404.2 (71.6-652.2)	<0.001
Use Dietary Supplement, n (%)	96 (14.9)	162 (26.1)	<0.001
Education, n (%)			<0.001
<High School or High School	473 (59.9)	143 (23.1)	
Some College, 4 Years of College	228 (28.9)	236 (38.2)	
Master, PhD, and Professional Degree	89 (11.3)	239 (38.7)	
Income, n (%)			<0.001
<$30,000	412 (48.5)	84 (13.8)	
$30,000-60,000	256 (30.2)	108 (17.7)	
≥$60,000	181 (21.3)	418 (68.5)	
Married or Living Like Married, n (%)	380 (43.2)	458 (74.0)	<0.001
Smoking, n (%)			<0.001
Current Smoker	219 (25.1)	48 (7.8)	
Yes, but Quit	295 (33.9)	247 (40.0)	
Alcohol Use, n (%)			<0.001
Yes, but Quit	223 (25.3)	92 (14.9)	
Currently Drink	445 (50.5)	483 (78.2)	
Family History, n (%)	141 (16.0)	117 (18.9)	0.15
PSA, median (IQR)			
Cases	7.5 (11.1)	5.0 (3.7)	<0.001
Controls	1.1 (1.7)	1.2 (1.6)	0.47
NCCN High Risk PCa[4], n (%)	105 (29.1)	35 (14.5)	<0.001
Gleason Score 4+3 or higher, n (%)	83 (24.2)	58 (25.2)	0.84

[1] Standard Deviation (SD)

[2] Interquartile Range (IQR)

[3] Vitamin DInternational Unit (IU, 1 IU = 0.025 μg)

[4] Based on National Comprehensive Cancer Network (NCCN) risk stratification for biochemical failure.
