# Supplementary material for: Race and BMI modify associations of calcium and vitamin D intake with prostate cancer
Source: BMC Cancer. 2017 Jan 19;17:64. doi: 10.1186/s12885-017-3060-8 (PMC5248493; doi:10.1186/s12885-017-3060-8)
Supplement: Additional file 2: Table S2. — Association of Dietary Calcium and Vitamin D Intake with Prostate Cancer in African Americans and European Americans. (PDF 278 kb) [file 12885_2017_3060_MOESM2_ESM.pdf]

Supplemental Table 2 Association of Dietary Calcium and Vitamin D Intake with Prostate Cancer in African Americans and European Americans

	Controls	Cases vs. Controls			NCCN High Risk vs. Controls			Gleason Score ≥4+3 vs. Controls		
			Unadjusted	Adjusted		Unadjusted	Adjusted		Unadjusted	Adjusted
	n (%)	n (%)	OR (95% C.I.)	OR (95% C.I.)	n (%)	OR (95% C.I.)	OR (95% C.I.)	n (%)	OR (95% C.I.)	OR (95% C.I.)
Dietary Calcium, mg/day										
African Americans										
Quartile 1 (<283.5)	132 (26.6)	110 (28.1)	1.00	1.00	25 (23.8)	1.00	1.00	16 (19.3)	1.00	1.00
Quartile 2 (283.5-504.4)	117 (23.5)	88 (22.5)	1.03 (0.71-1.49)	0.74 (0.47-1.18)	19 (18.1)	0.86 (0.45-1.64)	0.80 (0.35-1.82)	18 (21.7)	1.27 (0.62-2.60)	1.11 (0.46-2.69)
Quartile 3 (504.5-813.1)	120 (24.1)	85 (21.7)	0.95 (0.66-1.38)	0.83 (0.51-1.36)	20 (19.0)	0.88 (0.47-1.67)	1.51 (0.63-3.60)	16 (19.3)	1.10 (0.53-2.30)	1.77 (0.70-4.47)
Quartile 4 (>813.1)	128 (25.8)	108 (27.6)	1.08 (0.75-1.56)	0.83 (0.50-1.38)	41 (39.0)	1.69 (0.97-2.94)	1.78 (0.75-4.25)	33 (39.8)	2.13 (1.12-4.05)	2.40 (0.96-6.02)
P for Trend			0.84	0.89		0.02	**0.02**		0.01	**0.02**
European Americans										
Quartile 1 (<283.5)	86 (22.8)	56 (23.1)	1.00	1.00	6 (17.1)	1.00	1.00	10 (17.2)	1.00	1.00
Quartile 2 (283.5-504.4)	110 (29.1)	65 (26.9)	0.91 (0.58-1.43)	0.94 (0.57-1.53)	9 (25.7)	1.65 (0.85-3.21)	1.64 (0.49-5.50)	13 (22.4)	1.02 (0.43-2.43)	1.07 (0.43-2.69)
Quartile 3 (504.5-813.1)	106 (28.0)	68 (28.1)	0.99 (0.63-1.55)	1.00 (0.60-1.67)	12 (34.3)	1.64 (0.84-3.18)	2.68 (0.80-8.96)	20 (34.5)	1.62 (0.72-3.65)	1.73 (0.71-4.25)
Quartile 4 (>813.1)	76 (20.1)	53 (21.9)	0.78 (0.66-1.74)	1.10 (0.63-1.92)	8 (22.9)	1.79 (0.94-3.41)	1.87 (0.47-7.44)	15 (25.9)	1.70 (0.72-4.00)	1.84 (0.69-4.87)
P for Trend			0.64	0.94		0.42	0.89		0.14	0.30
P for Interaction (Dietary Calcium Intake x Race)				0.89			0.84			0.97
Dietary Vitamin D, IU/day										
African Americans										
Quartile 1 (<29.9)	116 (23.3)	80 (20.5)	1.00	1.00	16 (15.2)	1.00	1.00	13 (15.7)	1.00	1.00
Quartile 2 (29.9-71.4)	123 (24.7)	104 (26.6)	1.23 (0.83-1.80)	1.07 (0.67-1.72)	28 (26.7)	1.65 (0.85-3.21)	1.12 (0.49-2.54)	20 (24.1)	1.45 (0.69-3.05)	0.85 (0.34-2.12)
Quartile 3 (71.5-148.3)	124 (24.9)	96 (24.6)	1.12 (0.76-1.66)	1.00 (0.59-1.69)	28 (26.7)	1.64 (0.84-3.18)	0.91 (0.36-2.27)	23 (27.7)	1.66 (0.80-3.42)	0.90 (0.34-2.39)
Quartile 4 (>148.3)	134 (27.0)	111 (28.4)	1.20 (0.82-1.76)	0.92 (0.50-1.68)	33 (31.4)	1.79 (0.94-3.41)	0.67 (0.23-1.90)	27 (32.5)	1.80 (0.89-3.65)	0.68 (0.23-2.03)
P for Trend			0.56	0.61		0.19	0.29		0.15	0.58
European Americans										
Quartile 1 (<29.9)	106 (28.0)	71 (29.3)	1.00	1.00	8 (22.9)	1.00	1.00	13 (22.4)	1.00	1.00

Quartile 2 (29.9-71.4)	106 (28.0)	51 (21.1)	0.72 (0.46-1.13)	0.73 (0.44-1.20)	9 (25.7)	1.13 (0.42-3.03)	0.85 (0.27-2.61)	14 (24.1)	1.08 (0.48-2.40)	0.91 (0.38-2.14)
Quartile 3 (71.5-148.3)	86 (22.8)	71 (29.3)	1.23 (0.80-1.90)	1.18 (0.70-2.00)	12 (34.3)	1.85 (0.72-4.73)	1.43 (0.44-4.67)	18 (31.0)	1.71 (0.79-3.68)	1.18 (0.46-3.01)
Quartile 4 (>148.3)	80 (21.2)	49 (20.0)	0.91 (0.57-1.46)	0.97 (0.53-1.78)	6 (17.1)	0.99 (0.33-2.98)	0.71 (0.17-3.07)	13 (22.4)	1.33 (0.58-3.01)	1.06 (0.37-3.05)
P for Trend			0.80	0.59		0.95	0.64		0.43	0.82
P for Interaction (Dietary Vitamin D Intake x Race)				0.47			0.84			0.99
Supplemental Calcium, mg/day										
African Americans										
0	225 (45.3)	222 (56.8)	1.00	1.00	66 (62.9)	1.00	1.00	49 (59.0)	1.00	1.00
0-200	214 (43.1)	126 (32.2)	0.60 (0.45-0.80)	0.79 (0.51-1.23)	25 (23.8)	0.40 (0.24-0.66)	**0.41 (0.18-0.95)**	27 (32.5)	0.58 (0.35-0.96)	0.75 (0.33-1.70)
≥200	58 (11.7)	43 (11.0)	0.75 (0.49-1.16)	0.92 (0.51-1.65)	14 (13.3)	0.82 (0.43-1.57)	1.55 (0.56-4.25)	7 (8.4)	0.55 (0.24-1.29)	0.79 (0.25-2.54)
P for Trend			0.15	0.99		0.47	0.11		0.13	0.79
European Americans										
0	170 (45.0)	100 (41.3)	1.00	1.00	11 (31.4)	1.00	1.00	22 (37.9)	1.00	1.00
0-200	146 (38.6)	106 (43.8)	1.23 (0.87-1.75)	1.36 (0.84-2.19)	14 (40.0)	1.48 (0.65-3.37)	2.53 (0.79-8.09)	26 (44.8)	1.38 (0.75-2.53)	1.75 (0.76-4.00)
≥200	62 (16.4)	36 (14.9)	0.99 (0.61-1.59)	0.96 (0.53-1.75)	10 (28.6)	2.49 (1.01-6.16)	2.72 (0.74-10.03)	10 (17.2)	1.25 (0.56-2.78)	1.26 (0.44-3.56)
P for Trend			0.88	0.48		0.05	0.32		0.66	0.93
P for Interaction (Supplemental Calcium Intake x Race)				0.05			**0.01**			0.13
Supplemental Vitamin D, IU/day										
African Americans										
0	245 (49.3)	244 (62.4)	1.00	1.00	73 (69.5)	1.00	1.00	55 (66.3)	1.00	1.00
0-400	76 (15.3)	25 (6.4)	0.33 (0.20-0.54)	**0.54 (0.30-0.97)**	4 (3.8)	0.18 (0.06-0.50)	0.30 (0.08-1.09)	6 (7.2)	0.35 (0.15-0.85)	0.72 (0.25-2.05)
≥400	176 (35.4)	122 (31.2)	0.70 (0.52-0.93)	0.70 (0.48-1.01)	28 (26.7)	0.53 (0.33-0.86)	**0.34 (0.18-0.65)**	22 (26.5)	0.56 (0.33-0.95)	**0.44 (0.23-0.86)**
P for Trend			0.03	0.06		0.02	**0.001**		0.04	**0.02**
European Americans										
0	169 (44.7)	103 (42.6)	1.00	1.00	13 (37.1)	1.00	1.00	13 (37.1)	1.00	1.00
0-400	22 (5.8)	14 (5.8)	1.04 (0.51-2.13)	1.23 (0.55-2.75)	3 (8.6)	1.77 (0.47-6.71)	3.17 (0.64-15.59)	3 (8.6)	1.75 (0.60-5.08)	2.73 (0.81-9.22)
≥400	187 (49.5)	125 (51.7)	1.10 (0.79-1.53)	1.24 (0.84-1.82)	19 (54.3)	1.32 (0.63-2.76)	1.94 (0.75-5.05)	19 (54.3)	1.27 (0.71-2.29)	1.56 (0.77-3.17)

P for Trend	0.59	0.22	0.52	0.20	0.49	0.27
P for Interaction (Supplemental Vitamin D Intake x Race)		0.10		**0.03**		0.07

NOTE: Model adjusted for age, family history of PCa, BMI, education, smoking, alcohol use, and marital status, as well as mutually adjustment for total calcium or vitamin D intake. In the adjusted model, dietary and supplementary calcium and vitamin D intake simultaneously adjusted for each other. For the analysis of dietary and supplementary calcium intake, total vitamin D intake was adjusted for, while we adjusted for total calcium intake for the analysis of dietary and supplemental vitamin D intake. Significant association is shown with bolded type.
