# Supplementary material for: Race and BMI modify associations of calcium and vitamin D intake with prostate cancer
Source: BMC Cancer. 2017 Jan 19;17:64. doi: 10.1186/s12885-017-3060-8 (PMC5248493; doi:10.1186/s12885-017-3060-8)
Supplement: Additional file 3: Table S3. — Association of Dietary Calcium and Vitamin D Intake with Prostate Cancer in Stratified Analysis Based on Body Mass Index. (PDF 279 kb) [file 12885_2017_3060_MOESM3_ESM.pdf]

Supplemental Table 3 Association of Dietary Calcium and Vitamin D Intake with Prostate Cancer in Stratified Analysis Based on Body Mass Index

	Controls	Cases vs. Controls			NCCN High Risk vs. Controls			Gleason Score ≥4+3 vs. Controls		
			Unadjusted	Adjusted		Unadjusted	Adjusted		Unadjusted	Adjusted
	n (%)	n (%)	OR (95% C.I.)	OR (95% C.I.)	n (%)	OR (95% C.I.)	OR (95% C.I.)	n (%)	OR (95% C.I.)	OR (95% C.I.)
Dietary Calcium, mg/day										
BMI <Median (28.78)										
Quartile 1 (<283.5)	96 (22.2)	82 (23.6)	1.00	1.00	13 (16.3)	1.00	1.00	10 (13.9)	1.00	1.00
Quartile 2 (283.5-504.4)	115 (26.6)	88 (25.3)	0.90 (0.60-1.34)	0.88 (0.60-1.55)	20 (25.0)	1.28 (0.61-2.72)	1.43 (0.58-3.53)	19 (26.4)	1.59 (0.70-3.57)	1.51 (0.61-3.73)
Quartile 3 (504.5-813.1)	122 (28.2)	88 (25.3)	0.84 (0.57-1.26)	1.00 (0.62-1.63)	16 (20.0)	0.97 (0.44-2.11)	1.70 (0.64-4.47)	20 (27.8)	1.57 (0.70-3.52)	2.33 (0.92-5.89)
Quartile 4 (>813.1)	99 (22.9)	90 (25.9)	1.06 (0.71-1.60)	0.98 (0.58-1.65)	31 (38.8)	2.31 (1.14-4.68)	2.17 (0.83-5.69)	23 (31.9)	2.23 (1.01-4.93)	2.35 (0.91-6.12)
P for Trend			0.62	0.96		0.01	**0.049**		0.06	0.06
BMI ≥ Median (28.78)										
Quartile 1 (<283.5)	118 (25.3)	89 (27.3)	1.00	1.00	18 (25.7)	1.00	1.00	17 (22.1)	1.00	1.00
Quartile 2 (283.5-504.4)	119 (25.5)	76 (23.6)	0.85 (0.57-1.26)	0.82 (0.53-1.27)	11 (15.7)	0.61 (0.27-1.34)	0.54 (0.23-1.31)	13 (16.9)	0.76 (0.35-1.63)	0.65 (0.29-1.48)
Quartile 3 (504.5-813.1)	113 (24.2)	78 (23.3)	0.92 (0.61-1.36)	0.94 (0.59-1.51)	18 (25.7)	1.04 (0.52-2.11)	1.39 (0.58-3.36)	17 (22.1)	1.04 (0.51-2.15)	1.18 (0.53-2.65)
Quartile 4 (>813.1)	117 (25.1)	91 (25.8)	1.03 (0.70-1.52)	0.99 (0.62-1.59)	23 (32.9)	1.29 (0.66-2.51)	1.13 (0.46-2.75)	30 (39.0)	1.78 (0.93-3.40)	1.51 (0.69-3.30)
P for Trend			0.65	0.87		0.18	0.48		0.02	0.12
P for Interaction (Dietary Calcium Intake x BMI)				0.97			0.57			0.77
Dietary Vitamin D, IU/day										
BMI <Median (28.78)										
Quartile 1 (<29.9)	107 (24.8)	82 (23.6)	1.00	1.00	14 (17.5)	1.00	1.00	14 (19.4)	1.00	1.00
Quartile 2 (29.9-71.4)	101 (23.4)	87 (25.0)	1.12 (0.75-1.69)	1.03 (0.65-1.65)	22 (27.5)	1.67 (0.81-3.43)	1.45 (0.61-3.47)	19 (26.4)	1.44 (0.69-3.02)	1.19 (0.51-2.77)
Quartile 3 (71.5-148.3)	107 (24.8)	93 (26.7)	1.13 (0.76-1.69)	1.09 (0.67-1.79)	22 (27.5)	1.57 (0.76-3.23)	1.23 (0.49-3.08)	20 (27.8)	1.43 (0.69-2.98)	1.16 (0.48-2.83)
Quartile 4 (>148.3)	117 (27.1)	86 (24.7)	0.96 (0.64-1.43)	0.61 (0.34-1.08)	22 (27.5)	1.44 (0.70-2.95)	0.42 (0.14-1.26)	19 (26.4)	1.24 (0.59-2.60)	0.55 (0.20-1.55)
P for Trend			0.64	0.06		0.65	**0.03**		0.84	0.14
BMI ≥ Median (28.78)										
Quartile 1 (<29.9)	127 (27.2)	79 (23.7)	1.00	1.00	11 (15.7)	1.00	1.00	14 (18.2)	1.00	1.00

Quartile 2 (29.9-71.4)	124 (26.6)	76 (22.8)	0.99 (0.66-1.47)	0.94 (0.60-1.47)	15 (21.4)	1.40 (0.62-3.16)	1.15 (0.46-2.84)	15 (19.5)	1.10 (0.51-2.37)	0.83 (0.36-1.89)
Quartile 3 (71.5-148.3)	107 (22.9)	88 (26.3)	1.32 (0.89-1.97)	1.23 (0.75-2.04)	24 (34.3)	**2.59 (1.21-5.53)**	1.88 (0.69-5.11)	24 (31.2)	2.04 (1.01-4.13)	1.37 (0.55-3.39)
Quartile 4 (>148.3)	109 (23.3)	91 (27.2)	1.34 (0.90-1.99)	1.65 (0.93-2.94)	20 (28.6)	2.12 (0.97-4.62)	1.81 (0.46-1.72)	24 (31.2)	2.00 (0.99-4.05)	1.54 (0.57-4.18)
P for Trend			0.08	**0.03**		0.06	0.29		0.03	0.15
P for Interaction (Dietary Vitamin D Intake x BMI)				0.17			0.31			0.37
Supplemental Calcium, mg/day										
BMI <Median (28.78)										
0	188 (43.5)	188 (54.0)	1.00	1.00	47 (58.8)	1.00	1.00	41 (56.9)	1.00	1.00
0-200	169 (39.1)	118 (33.9)	0.70 (0.51-0.95)	0.80 (0.51-1.25)	25 (31.3)	0.59 (0.35-1.00)	0.74 (0.32-1.71)	25 (34.7)	0.68 (0.40-1.16)	0.96 (0.47-1.96)
≥200	75 (17.4)	42 (12.1)	0.56 (0.37-0.86)	0.62 (0.35-1.09)	8 (10.0)	0.43 (0.19-0.95)	0.82 (0.26-2.63)	6 (8.3)	0.37 (0.15-0.90)	0.50 (0.18-1.44)
P for Trend			0.009	0.13		0.04	0.62		0.03	0.15
BMI ≥ Median (28.78)										
0	222 (47.5)	161 (48.2)	1.00	1.00	36 (51.4)	1.00	1.00	34 (44.2)	1.00	1.00
0-200	192 (41.1)	125 (37.4)	0.90 (0.66-1.22)	1.05 (0.68-1.61)	17 (24.3)	0.55 (0.30-1.00)	0.83 (0.34-2.01)	29 (37.7)	0.99 (0.58-1.68)	1.22 (0.57-2.61)
≥200	53 (11.3)	48(14.4)	1.25 (0.80-1.94)	1.27 (0.73-2.23)	17 (24.3)	1.98 (1.03-3.79)	**3.33 (1.23-9.06)**	14 (18.2)	1.73 (0.86-3.44)	1.80 (0.70-4.62)
P for Trend			0.32	0.49		0.03	**0.005**		0.12	0.29
P for Interaction (Supplemental Calcium Intake x BMI)				0.05			**0.007**			**0.01**
Supplemental Vitamin D, IU/day										
BMI <Median (28.78)										
0	191 (44.2)	198 (56.9)	1.00	1.00	50 (62.5)	1.00	1.00	45 (62.5)	1.00	1.00
0-400	49 (11.3)	20 (5.7)	0.39 (0.23-0.69)	**0.52 (0.27-0.98)**	3 (3.8)	0.23 (0.07-0.78)	0.32 (0.08-1.22)	3 (4.3)	0.26 (0.08-0.87)	0.35 (0.10-1.27)
≥400	192 (44.4)	130 (37.4)	0.65 (0.49-0.88)	**0.69 (0.48-0.99)**	27 (33.8)	0.54 (0.32-0.89)	**0.44 (0.22-0.86)**	24 (33.3)	0.53 (0.31-0.91)	**0.43 (0.22-0.83)**
P for Trend			0.01	0.06		0.03	**0.02**		0.03	**0.02**
BMI ≥ Median (28.78)										
0	243 (52.0)	179 (53.6)	1.00	1.00	41 (58.6)	1.00	1.00	35 (45.5)	1.00	1.00
0-400	39 (8.4)	18 (5.4)	0.63 (0.35-1.13)	0.82 (0.43-1.56)	4 (5.7)	0.61 (0.21-1.79)	1.29 (0.40-4.21)	7 (9.1)	1.25 (0.52-3.00)	2.45 (0.93-6.44)
≥400	185 (39.6)	137 (41.0)	1.01 (0.75-1.35)	1.09 (0.78-1.54)	25 (35.7)	0.80 (0.47-1.37)	0.89 (0.46-1.72)	35 (45.5)	1.31 (0.79-2.18)	1.35 (0.74-2.45)

P for Trend	0.87	0.53	0.45	0.70	0.30	0.35
P for Interaction (Supplemental Vitamin D Intake x BMI)		0.16		0.22		**0.01**

NOTE: Model adjusted for age, family history of PCa, race/ethnicity, education, smoking, alcohol use, and marital status, as well as mutually adjustment for total calcium or vitamin D intake. In the adjusted model, dietary and supplementary calcium and vitamin D intake simultaneously adjusted for each other. For the analysis of dietary and supplementary calcium intake, total vitamin D intake was adjusted for, while we adjusted for total calcium intake for the analysis of dietary and supplemental vitamin D intake. Significant association is shown with bolded type.
