# Supplementary material for: Race and BMI modify associations of calcium and vitamin D intake with prostate cancer
Source: BMC Cancer. 2017 Jan 19;17:64. doi: 10.1186/s12885-017-3060-8 (PMC5248493; doi:10.1186/s12885-017-3060-8)
Supplement: Additional file 4: Table S4. — Association of Total Calcium and Vitamin D Intake with Prostate Cancer in Stratified Analysis Based on Body Mass Index in African Americans and European Americans. (PDF 263 kb) [file 12885_2017_3060_MOESM4_ESM.pdf]

Supplemental Table 4 Association of Total Calcium and Vitamin D Intake with Prostate Cancer in Stratified Analysis Based on Body Mass Index in African Americans and European Americans

	African American				European Americans			
	Controls	Cases	Unadjusted	Adjusted	Controls	Cases	Unadjusted	Adjusted
	n (%)	n (%)	OR (95% C.I.)	OR (95% C.I.)	n (%)	n (%)	OR (95% C.I.)	OR (95% C.I.)
Total Calcium, mg/day								
BMI <Median (27.8)								
Quartile 1 (<368.8)	54 (27.0)	57 (27.9)	1.00	1.00	32 (16.3)	28 (24.8)	1.00	1.00
Quartile 2 (368.8-616.0)	45 (22.5)	40 (19.6)	0.84 (0.48-1.48)	1.09 (0.51-2.34)	68 (34.7)	28 (24.8)	0.47 (0.24-0.92)	0.52 (0.24-1.10)
Quartile 3 (616.1-1033.3)	51 (25.5)	48 (23.5)	0.89 (0.52-1.53)	1.18 (0.55-2.51)	50 (25.5)	33 (29.2)	0.75 (0.39-1.48)	0.84 (0.38-1.86)
Quartile 4 (>1033.3)	50 (25.0)	59 (28.9)	1.12 (0.66-1.90)	2.23 (0.98-2.06)	46 (23.5)	24 (21.2)	0.60 (0.29-1.21)	0.60 (0.26-1.40)
P for Trend			0.56	**0.04**			0.53	0.44
BMI ≥ Median (27.8)								
Quartile 1 (<368.8)	73 (29.1)	51 (28.8)	1.00	1.00	45 (25.4)	25 (20.5)	1.00	1.00
Quartile 2 (368.8-616.0)	57 (22.7)	47 (26.3)	1.18 (0.70-2.00)	0.86 (0.45-1.65)	47 (26.6)	31 (25.4)	1.19 (0.61-2.31)	1.29 (0.62-2.67)
Quartile 3 (616.1-1033.3)	62 (24.7)	43 (24.0)	0.99 (0.59-1.68)	1.26 (0.63-2.52)	36 (20.3)	38 (31.1)	1.90 (0.97-3.71)	1.66 (0.77-3.57)
Quartile 4 (>1033.3)	59 (23.5)	38 (21.2)	0.92 (0.54-1.59)	0.65 (0.39-1.80)	49 (27.7)	28 (23.0)	1.03 (0.52-2.02)	0.87 (0.39-1.95)
P for Trend			0.62	0.86			0.89	0.54
P for Interaction (Total Calcium Intake x BMI)				0.47				0.24
Total Vitamin D, IU/day								
BMI <Median (27.8)								
Quartile 1 (<63.5)	42 (21.0)	57 (27.9)	1.00	1.00	37 (18.9)	27 (23.9)	1.00	1.00
Quartile 2 (63.5-239.2)	60 (30.0)	61 (29.9)	0.75 (0.44-1.28)	0.76 (0.36-1.60)	42 (21.4)	23 (20.4)	0.68 (0.36-1.28)	0.77 (0.33-1.78)
Quartile 3 (239.3-510.2)	51 (25.5)	55 (27.0)	0.80 (0.46-1.38)	0.68 (0.32-1.47)	47 (24.0)	26 (23.0)	0.87 (0.47-1.60)	0.95 (0.42-2.15)
Quartile 4 (>510.2)	47 (23.5)	31 (15.2)	0.49 (0.27-0.89)	**0.29 (0.12-0.72)**	70 (35.7)	37 (32.7)	0.61 (0.31-1.21)	0.87 (0.40-1.93)
P for Trend			0.04	**0.006**			0.47	0.99
BMI ≥ Median (27.8)								
Total Vitamin D, IU/day								

Quartile 1 (<63.5)	64 (25.5)	50 (27.9)	1.00	1.00	49 (27.7)	28 (23.0)	1.00	1.00
Quartile 2 (63.5-239.2)	64 (25.5)	51 (28.5)	1.02 (0.61-1.72)	0.99 (0.51-1.95)	37 (20.9)	21 (17.2)	0.68 (0.36-1.32)	0.87 (0.40-1.93)
Quartile 3 (239.3-510.2)	71 (28.3)	42 (23.5)	0.76 (0.45-1.29)	0.85 (0.43-1.70)	41 (23.2)	25 (20.5)	1.75 (0.92-3.30)	1.04 (0.49-2.22)
Quartile 4 (>510.2)	52 (20.7)	36 (20.1)	0.89 (0.50-1.56)	0.88 (0.40-1.91)	50 (28.2)	48 (39.3)	1.29 (0.67-2.49)	1.86 (0.89-3.90)
P for Trend			0.49	0.62			0.05	**0.03**
P for Interaction (Total Vitamin D Intake x BMI)				0.56				0.22

NOTE: Model adjusted for age, family history of PCa, education, smoking, alcohol use, and marital status as well as mutually adjustment for total calcium or vitamin D intake. Significant association is shown with bolded type.
