# Supplementary material for: Race and BMI modify associations of calcium and vitamin D intake with prostate cancer
Source: BMC Cancer. 2017 Jan 19;17:64. doi: 10.1186/s12885-017-3060-8 (PMC5248493; doi:10.1186/s12885-017-3060-8)
Supplement: Additional file 5: Table S5. — Institute of Medicine (IOM) Calcium and Vitamin D Dietary Reference Intakes and Prostate Cancer Stratified Based on Race. (PDF 262 kb) [file 12885_2017_3060_MOESM5_ESM.pdf]

Supplemental Table 5 Institute of Medicine (IOM) Calcium and Vitamin D Dietary Reference Intakes and Prostate Cancer Stratified Based on Race

	Controls	Cases vs. Controls			NCCN High Risk vs. Controls			Gleason Score ≥4+3 vs. Controls		
			Unadjusted	Adjusted		Unadjusted	Adjusted		Unadjusted	Adjusted
	n (%)	n (%)	OR (95% C.I.)	OR (95% C.I.)	n (%)	OR (95% C.I.)	OR (95% C.I.)	n (%)	OR (95% C.I.)	OR (95% C.I.)
Total Calcium, mg/day										
African Americans										
<800	331 (66.6)	247 (63.2)	1.00	1.00	57 (54.3)	1.00	1.00	44 (53.0)	1.00	1.00
800-1000	41 (8.2)	43 (11.0)	1.41 (0.89-2.22)	1.52 (0.88-2.62)	12 (11.4)	1.70 (0.84-3.43)	1.88 (0.81-4.41)	13 (15.7)	2.39 (1.19-4.80)	**2.81 (1.26-6.29)**
≥1000	125 (25.2)	101 (25.8)	1.08 (0.79-1.48)	1.10 (0.73-1.66)	36 (34.3)	1.67 (1.05-2.66)	1.76 (0.91-3.38)	26 (31.3)	1.57 (0.92-2.65)	1.82 (0.91-3.66)
P			0.34	0.32		0.06	0.13		0.03	**0.03**
European Americans										
<800	245 (64.8)	145 (59.9)	1.00	1.00	19 (54.3)	1.00	1.00	30 (51.7)	1.00	1.00
800-1000	31 (8.2)	38 (15.7)	2.07 (1.24-3.47)	**1.83 (1.05-3.18)**	3 (8.6)	1.25 (0.35-4.46)	1.04 (0.25-4.29)	9 (15.5)	2.37 (1.03-5.46)	2.02 (0.78-5.23)
≥1000	102 (27.0)	59 (24.4)	0.98 (0.67-1.43)	0.91 (0.60-1.40)	13 (37.1)	1.64 (0.78-3.45)	1.30 (0.54-3.13)	19 (32.8)	1.52 (0.82-2.83)	1.37 (0.68-2.74)
P			0.02	0.07		0.42	0.84		0.09	0.31
P for Interaction (Total Calcium Intake x Race)				0.76			0.82			0.93
Total Vitamin D, IU/day										
African Americans										
<400	304 (61.2)	253 (64.7)	1.00	1.00	69 (65.7)	1.00	1.00	54 (65.1)	1.00	1.00
400-600	113 (22.7)	92 (23.5)	0.98 (0.71-1.35)	0.92 (0.62-1.37)	29 (27.6)	1.13 (0.70-1.84)	0.82 (0.44-1.52)	22 (26.5)	1.10 (0.64-1.88)	1.05 (0.55-1.99)
≥600	80 (16.1)	46 (11.8)	0.69 (0.46-1.03)	**0.59 (0.36-0.98)**	7 (6.7)	0.39 (0.17-0.87)	**0.20 (0.07-0.57)**	7 (8.4)	0.49 (0.22-1.12)	**0.27 (0.09-0.80)**
P			0.19	0.13		0.05	**0.01**		0.20	**0.05**
European Americans										
<400	188 (49.7)	116 (47.9)	1.00	1.00	16 (45.7)	1.00	1.00	27 (46.6)	1.00	1.00
400-600	81 (21.4)	66 (27.3)	1.32 (0.89-1.97)	1.39 (0.90-2.15)	10 (28.6)	1.45 (0.63-3.33)	2.14 (0.80-5.73)	15 (25.9)	1.29 (0.65-2.55)	1.48 (0.69-3.17)
≥600	109 (28.8)	60 (24.8)	0.89 (0.60-1.32)	1.03 (0.67-1.60)	9 (25.7)	0.97 (0.42-2.27)	1.35 (0.49-3.73)	16 (27.6)	1.02 (0.53-1.98)	1.12 (0.52-2.42)
P			0.21	0.30		0.62	0.31		0.75	0.60

P for Interaction (Total Vitamin D Intake x Race)	0.41	0.17	0.24

NOTE: Model adjusted for age, family history of PCa, BMI, education, smoking, alcohol use, and marital status, as well as mutually adjustment for total calcium or vitamin D intake. The IOM EAR for calcium intake is 800 mg/day and for vitamin D intake is 400 IU/day. The RDA for calcium is 1,000 mg/day and for vitamin D is 600 IU/day. Significant association is shown with bolded type.
